# 4-{2-[(5-Chloro-2-hydroxy­benzyl­idene)amino]eth­yl}benzene­sulfonamide

**DOI:** 10.1107/S1600536808005084

**Published:** 2008-03-20

**Authors:** Zahid H. Chohan, Hazoor A. Shad, M. Nawaz Tahir, Islam Ullah Khan

**Affiliations:** a Department of Chemistry, Bahauddin Zakariya University, Multan-60800, Pakistan; b University of Sargodha, Department of Physics, Sargodha, Pakistan; c Government College University, Department of Chemistry, Lahore, Pakistan

## Abstract

In the mol­ecule of the title compound, C_15_H_15_ClN_2_O_3_S, the S atom adopts a distorted tetra­hedral coordination geometry with two O atoms, one N atom of the amide group and one C atom of the aromatic ring. An intra­molecular O—H⋯N hydrogen bond results in the formation of a planar six-membered ring, which is oriented with respect to the adjacent aromatic ring at a dihedral angle of 3.38 (11)°. Thus, the two rings are nearly coplanar. In the crystal structure, inter­molecular N—H⋯O hydrogen bonds link the mol­ecules into centrosymmetric dimers.

## Related literature

For general background, see: Supuran & Scozzafava (2001 ▶); Chohan & Shad (2007 ▶). For related literature, see: Chohan *et al.* (2008 ▶); Shad *et al.* (2008 ▶); Tahir *et al.* (2008 ▶); Li (2006 ▶).
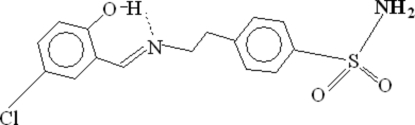


## Experimental

### 

#### Crystal data

C_15_H_15_ClN_2_O_3_S
*M* *_r_* = 338.80
Monoclinic, 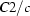

*a* = 21.069 (2) Å
*b* = 4.8125 (6) Å
*c* = 30.838 (3) Å
β = 99.942 (9)°
*V* = 3079.9 (6) Å^3^
*Z* = 8
Mo *K*α radiation
μ = 0.40 mm^−1^
*T* = 296 (2) K
0.22 × 0.18 × 0.14 mm

#### Data collection

Bruker Kappa APEXII CCD diffractometer
Absorption correction: multi-scan (*SADABS*; Bruker, 2005 ▶) *T*_min_ = 0.920, *T*_max_ = 0.940
16002 measured reflections
4067 independent reflections
1851 reflections with *I* > 3σ(*I*)
*R*_int_ = 0.060

#### Refinement

*R*[*F* ^2^ > 2σ(*F* ^2^)] = 0.046
*wR*(*F* ^2^) = 0.185
*S* = 1.02
4067 reflections
206 parameters
H atoms treated by a mixture of independent and constrained refinement
Δρ_max_ = 0.36 e Å^−3^
Δρ_min_ = −0.28 e Å^−3^

### 

Data collection: *APEX2* (Bruker, 2007 ▶); cell refinement: *APEX2*; data reduction: *SAINT* (Bruker, 2007 ▶); program(s) used to solve structure: *SHELXS97* (Sheldrick, 2008 ▶); program(s) used to refine structure: *SHELXL97* (Sheldrick, 2008 ▶); molecular graphics: *ORTEP-3 for Windows* (Farrugia, 1997 ▶) and *PLATON* (Spek, 2003 ▶); software used to prepare material for publication: *WinGX* (Farrugia, 1999 ▶) and *PLATON*.

## Supplementary Material

Crystal structure: contains datablocks global, I. DOI: 10.1107/S1600536808005084/hk2428sup1.cif

Structure factors: contains datablocks I. DOI: 10.1107/S1600536808005084/hk2428Isup2.hkl

Additional supplementary materials:  crystallographic information; 3D view; checkCIF report

## Figures and Tables

**Table:** 

S1—O2	1.430 (2)
S1—O3	1.423 (3)
S1—N2	1.616 (3)
S1—C13	1.752 (3)

**Table:** 

O2—S1—O3	118.15 (14)
O2—S1—N2	105.67 (16)
O2—S1—C13	109.21 (15)
O3—S1—N2	108.20 (17)
O3—S1—C13	108.29 (15)
N2—S1—C13	106.76 (15)

**Table 2:** Hydrogen-bond geometry (Å, °)

*D*—H⋯*A*	*D*—H	H⋯*A*	*D*⋯*A*	*D*—H⋯*A*
O1—H1⋯N1	0.82	1.83	2.554 (4)	147.00
N2—H2⋯O2^i^	0.77 (4)	2.30 (4)	3.016 (4)	156 (4)

Symmetry code: (i) 
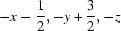
.

**Fig. 1.**
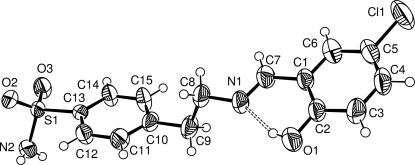
The molecular structure of the title molecule, with the atom-numbering scheme. Displacement ellipsoids are drawn at the 50% probability level.

**Fig. 2.**
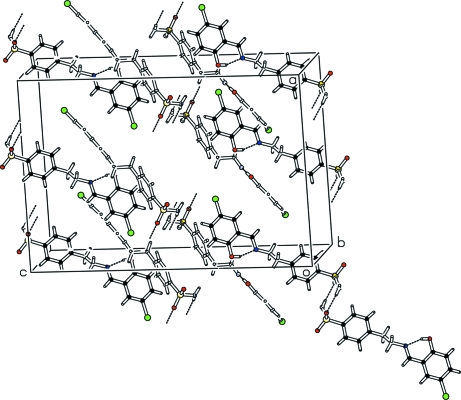
A packing diagram of (I). Hydrogen bonds are shown as dashed lines.

## supplementary crystallographic information

### Comment

The significance of sulfonamide was realised when sulfanilamide was first time reported as antibacterial drug. Later on, many sulfanilamide derivatives were synthesized, characterized and tested for antibacterial, anti-tumour, anti-carbonic anhydrase (Supuran & Scozzafava 2001), diuretic, hypoglycemic, anti-thyroid or protease inhibitory activity. Thus, sulfanilamide is performing a leading role for the development and expansion of all other types of medicinally important sulfonamides (Chohan & Shad, 2007).

The title compound, (I), is a reaction product of 5-chlorosalicylaldehyde and 4-(2-aminoethyl)benzenesulfonamide. The 4-(2-aminoethyl)benzenesulfonamide moiety is present in 4-[2-(3-ethyl-4-methyl-2-oxo-3-pyrrolidine-1-carboxamido)- ethyl]benzenesulfonamide (Li, 2006) and 5-chlorosalicylaldehyde exists in 4-[(5-chloro-2-hydroxybenzylidene)amino]-*N*-(3,4-dimethylisoxazol-5-yl)benzene- sulfonamide (Chohan *et al.*, 2008). In continuation of synthesizing Schiff base ligands of substituted halogen salicylaldehydes and various sulfonamides (Chohan *et al.*, 2008; Shad *et al.*, 2008; Tahir *et al.*, 2008), we report herein the crystal structure of the title compound, (I).

In the molecule of (I), S1 atom has a distorted tetrahedral coordination completed by the two O atoms, one N atom of amide group and one C atom of the adjacent aromatic ring B (C10—C15) (Table 1, Fig. 1). The two aromatic rings A (C1—C6) and B are connected by C=N—C—C group in a zigzag way, in which they are oriented at a dihedral angle of A/B = 23.95 (18)°. The intramolecular O—H···N hydrogen bond (Table 2) results in the formation of a planar six-membered ring C (O1/H1/N1/C7/C1/C2), which is oriented with respect to the aromatic rings at dihedral angles of A/C = 3.38 (11)° and B/C = 22.33 (13)°. So, rings A and C are also nearly coplanar.

In (I), C7—N1 [1.278 (5) Å] bond has double bond character. The C2=O1 [1.343 (5) Å] bond is a little longer than the corresponding value [1.328 (4) Å], as reported by Chohan *et al.* (2008). All other bonds in 5-chlorosalicylaldehyde moiety remain nearly same. The bond angles around S1 atom of sulfonamide group are a little smaller, having the range of 106.76 (15)° - 118.15 (14)°, compared to the values, in the range of 105.91 (13)°-119.68 (12)°, as reported by Li (2006).

In the crystal structure, intermolecular N—H···O hydrogen bonds (Table 2) link the molecules into centrosymmetric dimers (Fig. 2), in which they seem to be effective in the stabilization of the structure.

### Experimental

For the preparation of the title compound, an ethanol solution (15 ml) of 4-(2-aminoethyl)benzenesulfonamide (400.5 mg, 2 mmol) was added to an ethanol solution (10 ml) of 5-chlorosalicylaldehyde (313.1 mg, 2 mmol). The reaction mixture was refluxed for 3 h. The colour of the solution gradually changed from colorless to greenish yellow. The solution was cooled to room temperature, filtered and the volume was reduced to about one-third on the rotary evaporator. It was allowed to stand for 10 d, bright yellow crystals of the title compound were obtained (m.p. 441 K).

### Refinement

H atoms (for NH_2_) were located in a difference synthesis and refined isotropically [N—H = 0.77 (4) and 0.78 (4) Å; *U*_iso_(H) = 0.0767 and 0.0613 Å^2^]. The remaining H atoms were positioned geometrically, with O—H = 0.82 Å (for OH) and C—H = 0.93 and 0.97 Å for aromatic and methylene H atoms, respectively, and constrained to ride on their parent atoms, with *U*_iso_(H) = *xU*_eq_(C,*O*), where *x* = 1.5 for OH H and *x* = 1.2 for all other H atoms.

### Crystal data

**Table:**

C_15_H_15_ClN_2_O_3_S	*F*_000_ = 1408
*M**_r_* = 338.80	*D*_x_ = 1.461 Mg m^−^^3^
Monoclinic, *C*2/*c*	Melting point: 497 K
Hall symbol: -C 2yc	Mo *K*α radiation λ = 0.71073 Å
*a* = 21.069 (2) Å	Cell parameters from 2163 reflections
*b* = 4.8125 (6) Å	θ = 1.3–29.2º
*c* = 30.838 (3) Å	µ = 0.40 mm^−^^1^
β = 99.942 (9)º	*T* = 296 (2) K
*V* = 3079.9 (6) Å^3^	Prismatic, yellow
*Z* = 8	0.22 × 0.18 × 0.14 mm

### Data collection

**Table:**

Bruker KappaAPEXII CCD diffractometer	4067 independent reflections
Radiation source: fine-focus sealed tube	1851 reflections with *I* > 3σ(*I*)
Monochromator: graphite	*R*_int_ = 0.060
Detector resolution: 7.40 pixels mm^-1^	θ_max_ = 29.2º
*T* = 296(2) K	θ_min_ = 1.3º
ω scans	*h* = −28→28
Absorption correction: multi-scan(SADABS; Bruker, 2005)	*k* = −6→5
*T*_min_ = 0.920, *T*_max_ = 0.940	*l* = −41→42
16002 measured reflections	

### Refinement

**Table:**

Refinement on *F*^2^	Secondary atom site location: difference Fourier map
Least-squares matrix: full	Hydrogen site location: inferred from neighbouring sites
*R*[*F*^2^ > 2σ(*F*^2^)] = 0.047	H atoms treated by a mixture of independent and constrained refinement
*wR*(*F*^2^) = 0.185	*w* = 1/[σ^2^(*F*_o_^2^) + (0.0.0962*P*)^2^] where *P* = (*F*_o_^2^ + 2*F*_c_^2^)/3
*S* = 1.02	(Δ/σ)_max_ < 0.001
4067 reflections	Δρ_max_ = 0.36 e Å^−^^3^
206 parameters	Δρ_min_ = −0.28 e Å^−^^3^
Primary atom site location: structure-invariant direct methods	Extinction correction: none

### Special details

**Table:**

Geometry. Bond distances, angles *etc*. have been calculated using the rounded fractional coordinates. All su's are estimated from the variances of the (full) variance-covariance matrix. The cell e.s.d.'s are taken into account in the estimation of distances, angles and torsion angles
Refinement. Refinement of *F*^2^ against ALL reflections. The weighted *R*-factor *wR* and goodness of fit *S* are based on *F*^2^, conventional *R*-factors *R* are based on *F*, with *F* set to zero for negative *F*^2^. The threshold expression of *F*^2^ > σ(*F*^2^) is used only for calculating *R*-factors(gt) *etc*. and is not relevant to the choice of reflections for refinement. *R*-factors based on *F*^2^ are statistically about twice as large as those based on *F*, and *R*- factors based on ALL data will be even larger.

### Fractional atomic coordinates and isotropic or equivalent isotropic displacement parameters (Å^2^)

**Table:**

	*x*	*y*	*z*	*U*_iso_*/*U*_eq_	
Cl1	0.32293 (6)	0.3434 (3)	0.35291 (5)	0.1046 (5)	
S1	−0.14925 (4)	0.84474 (16)	−0.01326 (3)	0.0411 (3)	
O1	0.07158 (14)	−0.1064 (6)	0.27525 (10)	0.0724 (11)	
O2	−0.19990 (11)	1.0016 (4)	0.00047 (8)	0.0535 (9)	
O3	−0.10664 (12)	0.9849 (5)	−0.03704 (8)	0.0604 (9)	
N1	0.06373 (16)	0.2662 (6)	0.21557 (9)	0.0566 (10)	
N2	−0.18450 (17)	0.5984 (6)	−0.04399 (11)	0.0510 (11)	
C1	0.15618 (18)	0.2192 (8)	0.27152 (11)	0.0494 (11)	
C2	0.12970 (19)	−0.0013 (8)	0.29194 (12)	0.0526 (11)	
C3	0.1641 (2)	−0.1103 (9)	0.33014 (13)	0.0687 (16)	
C4	0.2227 (2)	−0.0078 (9)	0.34865 (13)	0.0677 (16)	
C5	0.24889 (19)	0.2059 (9)	0.32861 (14)	0.0663 (14)	
C6	0.2167 (2)	0.3195 (9)	0.29034 (13)	0.0655 (14)	
C7	0.1197 (2)	0.3508 (8)	0.23331 (12)	0.0590 (12)	
C8	0.0272 (2)	0.4227 (8)	0.17939 (12)	0.0622 (14)	
C9	0.0102 (2)	0.2436 (8)	0.13928 (12)	0.0653 (14)	
C10	−0.02942 (19)	0.3998 (7)	0.10209 (11)	0.0501 (11)	
C11	−0.09656 (19)	0.3851 (7)	0.09454 (12)	0.0556 (12)	
C12	−0.13287 (17)	0.5259 (7)	0.06036 (11)	0.0504 (11)	
C13	−0.10314 (14)	0.6854 (6)	0.03276 (10)	0.0355 (9)	
C14	−0.03664 (16)	0.7064 (8)	0.03996 (12)	0.0538 (11)	
C15	−0.00076 (18)	0.5662 (9)	0.07457 (13)	0.0625 (13)	
H1	0.05445	−0.00964	0.25463	0.1087*	
H2	−0.212 (2)	0.526 (9)	−0.0349 (15)	0.0767*	
H2A	−0.160 (2)	0.495 (9)	−0.0511 (14)	0.0613*	
H3	0.14678	−0.25811	0.34366	0.0825*	
H4	0.24468	−0.08276	0.37477	0.0812*	
H6	0.23528	0.46403	0.27690	0.0786*	
H7	0.13739	0.50280	0.22110	0.0710*	
H8A	0.05252	0.58026	0.17261	0.0746*	
H8B	−0.01190	0.49318	0.18800	0.0746*	
H9A	0.04945	0.17819	0.13017	0.0782*	
H9B	−0.01370	0.08266	0.14642	0.0782*	
H11	−0.11706	0.27761	0.11306	0.0667*	
H12	−0.17758	0.51368	0.05583	0.0607*	
H14	−0.01635	0.81493	0.02147	0.0647*	
H15	0.04386	0.58389	0.07956	0.0747*	

### Atomic displacement parameters (Å^2^)

**Table:**

	*U*^11^	*U*^22^	*U*^33^	*U*^12^	*U*^13^	*U*^23^
Cl1	0.0648 (7)	0.1294 (11)	0.1019 (10)	0.0029 (8)	−0.0357 (7)	−0.0252 (8)
S1	0.0365 (4)	0.0370 (4)	0.0461 (5)	−0.0006 (4)	−0.0030 (3)	0.0111 (4)
O1	0.0634 (17)	0.076 (2)	0.0685 (19)	−0.0105 (16)	−0.0151 (14)	0.0163 (14)
O2	0.0434 (14)	0.0394 (12)	0.0741 (18)	0.0078 (11)	0.0002 (13)	0.0034 (12)
O3	0.0485 (14)	0.0685 (15)	0.0621 (16)	−0.0062 (14)	0.0036 (13)	0.0313 (13)
N1	0.0563 (18)	0.0628 (18)	0.0439 (17)	0.0072 (17)	−0.0104 (15)	0.0043 (14)
N2	0.054 (2)	0.0502 (19)	0.0440 (18)	−0.0025 (15)	−0.0054 (15)	0.0014 (14)
C1	0.0497 (19)	0.058 (2)	0.0367 (18)	0.0091 (18)	−0.0034 (16)	−0.0007 (16)
C2	0.057 (2)	0.057 (2)	0.0391 (19)	0.012 (2)	−0.0050 (17)	−0.0024 (16)
C3	0.078 (3)	0.069 (3)	0.053 (2)	0.012 (2)	−0.006 (2)	0.010 (2)
C4	0.074 (3)	0.076 (3)	0.044 (2)	0.024 (3)	−0.015 (2)	−0.002 (2)
C5	0.051 (2)	0.086 (3)	0.054 (2)	0.015 (2)	−0.0134 (19)	−0.023 (2)
C6	0.057 (2)	0.081 (3)	0.053 (2)	0.003 (2)	−0.0063 (19)	0.001 (2)
C7	0.061 (2)	0.066 (2)	0.045 (2)	0.001 (2)	−0.0047 (18)	0.0111 (18)
C8	0.069 (3)	0.055 (2)	0.055 (2)	0.014 (2)	−0.011 (2)	0.0057 (18)
C9	0.083 (3)	0.059 (2)	0.045 (2)	0.021 (2)	−0.014 (2)	0.0039 (18)
C10	0.062 (2)	0.046 (2)	0.0371 (18)	0.0077 (19)	−0.0063 (17)	0.0017 (15)
C11	0.058 (2)	0.061 (2)	0.045 (2)	−0.005 (2)	0.0013 (18)	0.0167 (17)
C12	0.0418 (19)	0.061 (2)	0.045 (2)	−0.0051 (18)	−0.0017 (16)	0.0101 (17)
C13	0.0348 (15)	0.0331 (16)	0.0366 (17)	−0.0014 (14)	0.0003 (13)	0.0030 (13)
C14	0.0354 (17)	0.070 (2)	0.054 (2)	−0.0030 (19)	0.0025 (17)	0.0195 (19)
C15	0.0372 (18)	0.088 (3)	0.058 (2)	0.009 (2)	−0.0039 (17)	0.012 (2)

### Geometric parameters (Å, °)

**Table:**

Cl1—C5	1.741 (4)	C9—C10	1.499 (5)
S1—O2	1.430 (2)	C10—C15	1.380 (5)
S1—O3	1.423 (3)	C10—C11	1.395 (6)
S1—N2	1.616 (3)	C11—C12	1.370 (5)
S1—C13	1.752 (3)	C12—C13	1.375 (5)
O1—C2	1.343 (5)	C13—C14	1.384 (5)
O1—H1	0.8200	C14—C15	1.374 (5)
N1—C7	1.278 (5)	C3—H3	0.9300
N1—C8	1.452 (5)	C4—H4	0.9300
N2—H2	0.77 (4)	C6—H6	0.9300
N2—H2A	0.78 (4)	C7—H7	0.9300
C1—C7	1.438 (5)	C8—H8A	0.9700
C1—C2	1.398 (5)	C8—H8B	0.9700
C1—C6	1.393 (6)	C9—H9A	0.9700
C2—C3	1.376 (6)	C9—H9B	0.9700
C3—C4	1.360 (6)	C11—H11	0.9300
C4—C5	1.365 (6)	C12—H12	0.9300
C5—C6	1.369 (6)	C14—H14	0.9300
C8—C9	1.499 (5)	C15—H15	0.9300
			
O2—S1—O3	118.15 (14)	S1—C13—C12	119.8 (2)
O2—S1—N2	105.67 (16)	C12—C13—C14	119.9 (3)
O2—S1—C13	109.21 (15)	S1—C13—C14	120.2 (2)
O3—S1—N2	108.20 (17)	C13—C14—C15	119.7 (3)
O3—S1—C13	108.29 (15)	C10—C15—C14	121.5 (4)
N2—S1—C13	106.76 (15)	C2—C3—H3	119.00
C2—O1—H1	109.00	C4—C3—H3	119.00
C7—N1—C8	119.4 (3)	C3—C4—H4	120.00
S1—N2—H2	115 (3)	C5—C4—H4	120.00
S1—N2—H2A	112 (3)	C1—C6—H6	120.00
H2—N2—H2A	113 (5)	C5—C6—H6	120.00
C2—C1—C7	120.3 (3)	N1—C7—H7	119.00
C6—C1—C7	120.7 (4)	C1—C7—H7	119.00
C2—C1—C6	118.9 (3)	N1—C8—H8A	109.00
O1—C2—C1	121.4 (3)	N1—C8—H8B	110.00
O1—C2—C3	119.7 (4)	C9—C8—H8A	109.00
C1—C2—C3	118.9 (4)	C9—C8—H8B	110.00
C2—C3—C4	121.8 (4)	H8A—C8—H8B	108.00
C3—C4—C5	119.5 (4)	C8—C9—H9A	109.00
Cl1—C5—C4	119.6 (3)	C8—C9—H9B	109.00
Cl1—C5—C6	119.5 (3)	C10—C9—H9A	109.00
C4—C5—C6	120.8 (4)	C10—C9—H9B	109.00
C1—C6—C5	120.2 (4)	H9A—C9—H9B	108.00
N1—C7—C1	122.3 (4)	C10—C11—H11	119.00
N1—C8—C9	110.8 (3)	C12—C11—H11	119.00
C8—C9—C10	111.4 (3)	C11—C12—H12	120.00
C9—C10—C15	121.1 (4)	C13—C12—H12	120.00
C11—C10—C15	117.8 (3)	C13—C14—H14	120.00
C9—C10—C11	121.1 (3)	C15—C14—H14	120.00
C10—C11—C12	121.3 (3)	C10—C15—H15	119.00
C11—C12—C13	119.9 (3)	C14—C15—H15	119.00
			
O2—S1—C13—C12	52.8 (3)	C2—C3—C4—C5	−1.1 (6)
O2—S1—C13—C14	−131.0 (3)	C3—C4—C5—Cl1	178.4 (3)
O3—S1—C13—C12	−177.3 (3)	C3—C4—C5—C6	0.5 (6)
O3—S1—C13—C14	−1.1 (3)	Cl1—C5—C6—C1	−177.3 (3)
N2—S1—C13—C12	−61.0 (3)	C4—C5—C6—C1	0.6 (6)
N2—S1—C13—C14	115.2 (3)	N1—C8—C9—C10	178.1 (3)
C8—N1—C7—C1	174.7 (3)	C8—C9—C10—C11	−95.0 (4)
C7—N1—C8—C9	122.1 (4)	C8—C9—C10—C15	84.1 (5)
C6—C1—C2—O1	−180.0 (4)	C9—C10—C11—C12	−179.5 (3)
C6—C1—C2—C3	0.5 (6)	C15—C10—C11—C12	1.5 (5)
C7—C1—C2—O1	3.9 (6)	C9—C10—C15—C14	178.9 (4)
C7—C1—C2—C3	−175.7 (4)	C11—C10—C15—C14	−2.0 (6)
C2—C1—C6—C5	−1.1 (6)	C10—C11—C12—C13	0.0 (5)
C7—C1—C6—C5	175.1 (4)	C11—C12—C13—S1	175.4 (3)
C2—C1—C7—N1	−3.0 (6)	C11—C12—C13—C14	−0.9 (5)
C6—C1—C7—N1	−179.1 (4)	S1—C13—C14—C15	−175.9 (3)
O1—C2—C3—C4	−179.0 (4)	C12—C13—C14—C15	0.3 (5)
C1—C2—C3—C4	0.6 (6)	C13—C14—C15—C10	1.2 (6)

### Hydrogen-bond geometry (Å, °)

**Table:**

*D*—H···*A*	*D*—H	H···*A*	*D*···*A*	*D*—H···*A*
O1—H1···N1	0.8200	1.8300	2.554 (4)	147.00
N2—H2···O2^i^	0.77 (4)	2.30 (4)	3.016 (4)	156 (4)

Symmetry codes: (i) −*x*−1/2, −*y*+3/2, −*z*.
